# A reversible case of rapidly progressive dementia—Hypercalcemia due to hyperparathyroidism

**DOI:** 10.1002/ccr3.2564

**Published:** 2019-11-24

**Authors:** Josiana de Oliveira Martins Duarte, Paula Maria Lobato Pestana Pereira, João Nuno Gamito Lopes, Ana Teresa Loução Goes, David Campoamor Durán, Ana Sofia Gonçalves Nunes Sobral, Henrique José Barrelas Rita, José António de Sousa e Costa

**Affiliations:** ^1^ Internal Medicine Service Hospital do Litoral Alentejano Santiago do Cacem Portugal; ^2^ Intensive Care Unit Hospital do Litoral Alentejano Santiago do Cacem Portugal

**Keywords:** hypercalcemia, Hyperparathyroidism, rapidly progressive dementia, reversible dementia

## Abstract

With an increasingly aging population, it is of extreme importance to exclude potentially reversible dementias, such as hyperparathyroidism with hypercalcemia, in the differential diagnosis of a rapidly progressive dementia. According to literature, this entity is undervalued and it is highly relevant to be aware of it.

## Graphical abstract

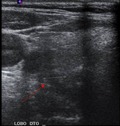

## 1 INTRODUCTION

The expression rapidly progressive dementia (RPD) is used to illustrate cases of neurocognitive impairment with an evolution which usually ranges between weeks and months.

Primary hyperparathyroidism (PTHP) may manifest as a rapidly progressive dementia with many neuropsychiatric symptoms, ranging from anxiety, affective disorders, personality changes, sleep disorders and cognitive impairment to severe psychotic conditions, coma and even death.

The hyperparathyroidism courses with hypercalcemia, hypophosphatemia and high levels of PTH (parathyroid hormone), in patients with normal renal function. Around 80% of cases, this change is caused by a solitary parathyroid adenoma (usually benign), resulting in increased parathyroid hormone (PTH) production and consequent increase in serum calcium and decrease of serum phosphorus.

Although PHTP is mainly associated with the classical clinical picture ("painful bones, kidney stones, abdominal groans, and psychic moans"),^2^ in the elderly, the clinical picture includes a very variable range of symptoms.

The initial presentation of PHTP as dementia is relatively rare, but has a higher incidence in older individuals. The ease of detection of this illness and the striking response to surgical intervention argue for a vigilant search of this diagnosis in the elderly patients.

## 2 CASE DESCRIPTION

We report a case of 73‐years‐old female admitted to our hospital due to prostration and markedly limitation in activities of daily living in the past two days.

Initial evaluation revealed a Glasgow Coma Scale of 13 points, without focal neurological changes, high blood pressure (BP 165/78 mm Hg), signs of dehydration, fever (38.7°C), and purulent effusion from the right hear. Otoscopy showed opaque yellow tympanic membrane at right ear.

Her altered mental status was initially interpreted in context of a Systemic Inflammatory Response Syndrome (SIRS) due to a suppurative otitis media and she was hospitalized to comply intravenous antibiotics and fluids (due to prostration she was unable to perform oral antibiotic treatment or hydration).

Her past medical history included the following: hypertension and hypercholesterolemia well controlled with diet (without any medication), type 2 Diabetes well controlled with metformin (1 g twice a day, with a glycated hemoglobin of 6%) and dementia diagnosed < 1 year ago and medicated with Zolpidem, Olanzapine, and Donepezil by a Psychiatrist.

During hospitalization, there was resolution of the infection and of some electrolyte changes, namely hypernatremia and hypokalemia, but we noted marked behavioral disturbances. This included the following: apathy (with little improvement after resolution of infection), poor discourse, disturbances of sleep (insomnia during the night, excessive sleep during daytime) and spatial awkwardness, without motor or sensitive deficits.

At this time, we asked relatives a more detailed history of her mental status. They described a less than twelve‐month cognitive impairment with increasing social isolation, some memory loss, apathy, anhedonia and decreased ability to care for herself (hygiene and nutrition). Because of these disturbances, her family physician sent her to a psychiatric consult but the relatives describes few improvements with medication prescribed. They also narrate that in the last 3 weeks before the hospitalization there was a rapidly progression to avolition, poor discourse, clinophilia, and evolution to total dependence of daily living activities.

With this history and behavioral disturbances observed during hospitalization, we hypothesized a rapidly progressive dementia and we started a differential diagnostic gait.

Simple biochemical and hematological laboratory tests were within the normal range, apart from a raised serum calcium, with a total corrected calcium for albumin 13.5 mg/dL (normal range 8.5‐10.5 mg/dL). After finding hypercalcemia, parathyroid hormone (PTH) was requested, with an increased value of 276.30 pg/mL (normal range 12.00‐88.00 pg/mL) and low serum phosphate value of 2.1 mg/dL (normal range 2.5‐4.6 mg/dL).

As part of the differential diagnosis, a cranial CT scan was also requested, showing a mild chronic ischemic microangiopathic leukoencephalopathy. Electrocardiogram and X‐ray of the thorax were normal.

There was a slowly response to therapy, namely fluid therapy, performed during hospitalization and patient was discharged home oriented to Internal Medicine consult for review and completion of the study.

**Figure 1:**
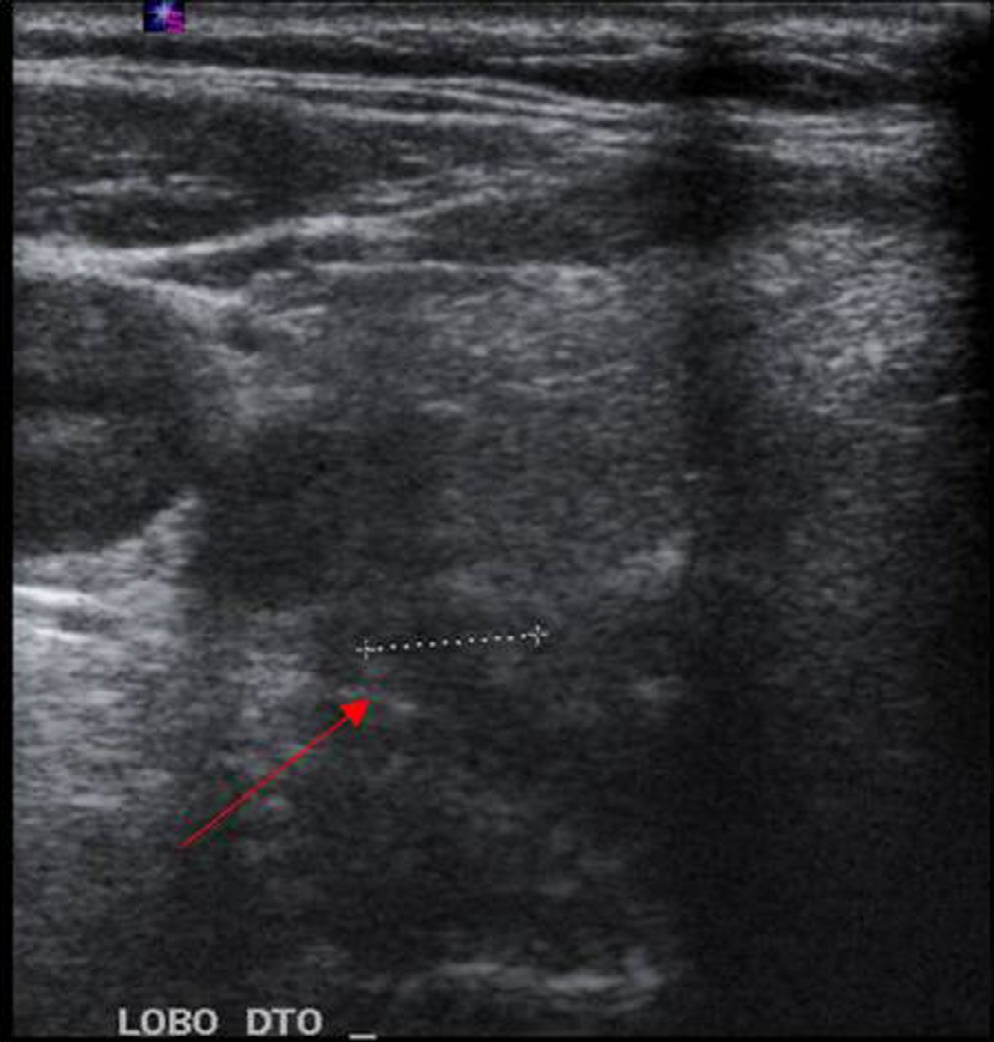
Hypoechoic node of the right parathyroid

With the triad of hypercalcemia, hypophosphatemia, and increased PTH, we suspected of primary hyperparathyroidism and we request an ultrasound of thyroid and parathyroid glands. This revealed a hypoechoic node of the right parathyroid probably related to an adenoma (Figure 1).

Although ultrasound is not the best imaging modality for localizing parathyroid adenomas, sestamibi scan was not performed because it was not available at our hospital.

An elevated urinary calcium of > 300 mg/day excluded familial hypocalciuric hypercalcemia, and the research to exclude the secondary causes of hyperparathyroidism was negative (namely normal renal function and normal levels of vitamin D).

As there were no other symptoms of hyperparathyroidism, particularly gastrointestinal or bone symptoms, during hospitalization, hypercalcaemia was treated initially with saline infusion to replace dehydration. Once the intravascular volume was restored she was also treated with loop diuretics (furosemide) and with bisphosphonates (zoledronic acid) until surgery.

One month later, she had another internal medicine consult and there was some clinical improvement and progressive recovery of independence of a few activities of daily living. Laboratory test revealed a normal serum calcium corrected for albumin 10.6 mg/dL, and she was then referred to Surgery consult.

Another month later, she was submitted to a subtotal parathyroidectomy. During this procedure, an intraoperative parathyroid hormone assay was performed showing a level fail after resection and confirming that no other abnormal glands were present.

At postoperative reevaluation consultation (2 weeks later), she was pretty well and showing signs of recovery of cognitive impairment, including taking care of her grandchildren again.

## 3 DISCUSSION

We report an exceptional case of a 73‐year‐old woman with primary hyperparathyroidism presenting as dementia, which improved after initial treatment with medical measures, namely fluid replacement and then loop diuretics and zoledronic acid. After that, they performed a definitive treatment with a subtotal parathyroidectomy.

Apathy, depression, and cognitive impairment are common presentations in several geriatric conditions.^3^ Primary hyperparathyroidism, though common among geriatric population, may be missed, and the symptoms may be prematurely diagnosed as Alzheimer's disease.^1^ Since hyperparathyroidism has a good potential for reversal of symptoms by medical or surgical options, a meticulous workup of all possible differential diagnosis for dementia is crucial.

In Table 1, we summarized the few similarly cases with dementia as a presenting symptom of hypercalcemia.

**Table 1:** Reported cases in the literature of hypercalcemia presenting as dementia

	Primary Author	Journal	PA	Age	Sex	PPP	T	Clinical	CA	PTH	ST
1	C. Ann McDonald	International Journal of Geriatric Psychiatry	2002	89	F	No	12	Decreased ability to care herself; disorientation; depression; memory loss	10.96	6.92	Yes
2	Lea C. Watson	Psychosomatics	2002	63	M	No	2	“not been himself”; “ getting lost”; “talking strange”; psychotic symptoms	10.8	127	Yes
3	Sokratis G. Papageorgiou	Clinical Neurology and Neurosurgery	2008	76	F	No	24	Withdrawal; Clinophilia; apathy; Deficit in concentration and communication	11.7	7.5	Yes
4	Harmit Singh	Psychiatry	2010	76	M	D AD	12	Disorientation; forgetfulness	10.8	109	No
5	S. Rocha	21st Meeting of European Neurological Society	2011	71	F	No	3	Disorientation; short stepped gait; generalized hyperreflexia	Slight HCA	1270	No
6	Marlena Zajączkowska	Med Sci Rev	2015	65	M	No	3	Impairment of cognitive function and bizarre, disorganized behavior	14.96	74.46	Yes

Abbreviations: AD, Alzheimer's disease; CA, Total Calcium corrected (mg/dL); D, Depression; F, Female; HCA, Hypercalcemia; M, Male; PA, Publication year; PPP, Previous Psychiatric pathology; PTH, Parathormone value (pg/mL); ST, Surgical treatment; T, Installation Time (months).

The relevance of this case is the need for being alert, considering primary hyperparathyroidism in the differential diagnosis of the elderly patient with neuropsychiatric disorders of recent onset (rapidly progressive dementia). As the screening of this disease is relatively easy and there is a high potential of reversibility, we should rule out this entity in these patients. Therefore, routine determination of serum calcium, followed by determination of parathyroid hormone (if elevated serum calcium levels), may be of great clinical utility in these cases.

Surgical excision of the abnormal parathyroid gland offers the only permanent, curative treatment for the primary hyperparathyroidism.^4^, ^5^

There is some controversy about the optimal management of asymptomatic patients and patients who do not undergo to surgery require long‐term monitoring.^6^, ^7^ This include, assessment of overt signs and symptoms of hyperparathyroidism annually, annual serum calcium and creatinine testing and bone mineral density (spine, hip, and forearm) every 1‐2 years.^5^

## AUTHOR CONTRIBUTION

JD: assumed the responsibility for the publication, making sure that the data are accurate, that all deserving authors have been credited; responsible for data collection and analysis; monitoring the progress of the disease and responsible for the literature review and final approval, submitting revisions and final version, and communicating with editors. PP: co‐responsible for data collection and contributed greatly to the case presentation and discussion section. JL: co‐responsible for data collection, contributed greatly to the case presentation. AG: co‐responsible for data collection, contributed greatly to the case presentation. DC: co‐responsible for data collection, contributed greatly to the case presentation. SS: co‐responsible for data collection, contributed greatly to the case presentation. HR: responsible for the revision of the article. JSC: responsible for the revision of the article.
